# Low-Fidelity Canthotomy Model Use in Resident Education: The Learner’s Perspective

**DOI:** 10.7759/cureus.12847

**Published:** 2021-01-21

**Authors:** Yasmany Cartaya, Andrew Little, Camilo M Mohar

**Affiliations:** 1 Emergency Medicine, AdventHealth East Orlando, Orlando, USA

**Keywords:** lateral canthotomy, medical education, simulation, training, low fidelity, high risk, likert scale, t test, synthetic model

## Abstract

Lateral canthotomy is a low incidence but high-risk procedure that is expected to be within the scope of practice for every emergency department physician. Due to the low incidence of this procedure, training residents may prove difficult and costly. The purpose of this study was to evaluate a synthetic, low-fidelity, low-cost, eye model to train and improve confidence in performing a lateral canthotomy. This is the first study that we know of that uses a 5 point Likert scale, two-sample t-test to assess the value of using such a model for lateral canthotomy training. Our results showed that using such a model for the training of lateral canthotomy did in fact improve the competence and confidence of performing this procedure.

## Introduction

Lateral canthotomy is a time-sensitive procedure required to be mastered by residents in emergency medicine (EM) as it is organ saving [1]. It is in the scope of practice for every EM physician. The eye is surrounded by a rigid, bony cone-shaped socket, consisting of various bones. Due to this enclosed space, the orbit is sensitive to pressure and gives little way when filled, compressing the soft tissue structures within it all the while increasing intraocular pressure. This causes a tight orbit referred to as orbital compartment syndrome (OCS) [2,3]. A lateral canthotomy is required immediately to relieve such pressure, in order to prevent permanent vision damage [4].

OCS can occur in various different ways such as expanding spontaneous retrobulbar hemorrhage, infection, fluid from resuscitation in burn victims, or orbital emphysema after blowing one’s nose with nasal bone fractures. In one study, OCS had an incidence of 0.088%, 16 individuals out of 18 thousand craniomaxillofacial emergent cases in a 3.5 year period, mostly a sequela of trauma [5]. The low incidence of experiencing such pathology while in residency makes bedside training unlikely. Historical training with animal models (pigs, dogs, cats, etc.), is ethically questionable and cost prohibitive. Although these animal models may simulate the tactile feel of a human eye, learners are not afforded what is needed to master any skill--repetitiveness [1]. This has led to educators moving to synthetic models, which may become cost-prohibitive. A viable alternative method needs to be investigated and validated.

Alternative methods used for the training of residents have been published as “homemade” low-fidelity eye models. However, few of these models have been validated in their effectiveness for training. One of these low-fidelity “homemade” models, described by Kong et al. contributes a low-cost alternative as well as a novel approach in teaching lateral canthotomy [1]. The purpose of this brief research report explains how the use of “homemade" models for lateral canthotomy was received by residents, and fared in improving the confidence needed to execute such a procedure. This study will help improve our understanding, regarding the effectiveness of using such models in residency training.

## Materials and methods

The researchers searched the currently available medical literature for “homemade” lateral canthotomy models, finding two separate innovations, with the researchers choosing the one with both written and pictorial instructions for their creation (Kong et al.). Supplies were purchased to create the low-fidelity, low-cost model. During a scheduled simulation day at a community hospital residency program, the model was used for the training of lateral canthotomy. This was performed in the simulation lab at the community hospital, where residents gathered to first see a demonstration of the lateral canthotomy performed on the model and then formed a queue to perform the procedure. Eighteen EM learners of various postgraduate year (PGY) levels used the trainer and completed a short survey before and after the completion of the procedure. It included information such as what was their training level, if the resident had completed a lateral canthotomy previously (with a patient or in simulation), if they felt the model helped them feel more comfortable, with performing a lateral canthotomy, and their thoughts on the model.

**Figure 1:**
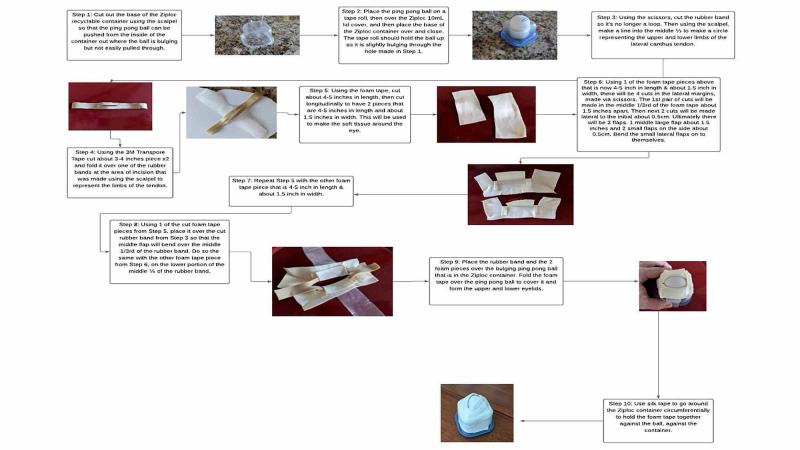
Low-fidelity Lateral Canthotomy Assembly

Items required for the eye model included ping pong ball, 10 mL Ziploc recyclable container, and lid, pressure foam tape, 3M Transpore tape (3M, St. Paul, MN, USA), silk tape, tape roll, circular standard size rubber band, scissors, and scalpel. There is a flowchart on the creation of the model by Kong et al. (Figure 1) [1].

## Results

**Figure 2:**
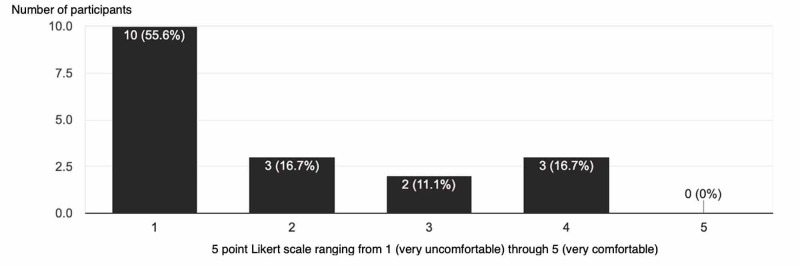
Preprocedure Questionnaire Y axis is number of participants X axis is a 5 point Likert scale ranging from 1 (very uncomfortable) through 5 (very comfortable)

**Figure 3:**
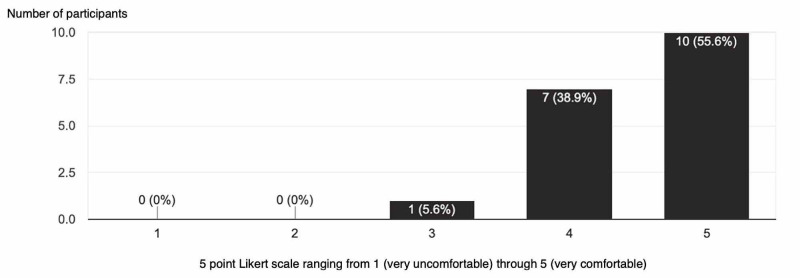
Postprocedure Questionnaire Y axis is number of participants X axis is a 5 point Likert scale ranging from 1 (very uncomfortable) through 5 (very comfortable)

After the procedure was performed, participants were asked to scan a QR code that would link to a questionnaire. The questionnaire would consist of what PGY level they were, have they performed a lateral canthotomy prior to the lab, their comfort level on performing the procedure prior to and after the lab. Out of eighteen participants, only one PGY3 resident had performed such a procedure on a living patient. The assessment of the comfort level for doing a procedure before and after the teaching lab was gauged using a 5 point Likert scale ranging from 1 (very uncomfortable) through 5 (very comfortable). Prior to the procedure lab, participants had a mean comfort level of 1.9, and after the procedure lab, the comfort level rose to 4.5 (Figures 2-3). When both mean values are compared using a two-sample t-test and using a 95% confidence interval. results revealed a significant improvement in comfort level with a p-value of <0.0001. All learners felt the model was a good representation of eye anatomy and the overall procedure was less than $3 for each model. 

## Discussion

A lateral canthotomy can greatly improve morbidity, in the event of OCS. The procedure is organ saving and is crucial for EM residents to master. Due to the low incidence of OCS, training in vivo is limited [2-6]. Animal models provide limited opportunity for procedural mastery and like prefabricated commercial models, it can be cost-prohibitive [1]. However, this low-fidelity “homemade” model provides residents the ability to familiarize themselves with each step without the pressure of wasting valuable specimens or equipment. Our study adds strength to the validity of this model in the training of a lateral canthotomy via the assessment of our surveys. Benefits of this model include low cost and ease of reproduction. We deduce that this model is an excellent alternative in the training of lateral canthotomy.

This study is not without its limitations. First, this study was performed at a single training site and future investigators would be wise to expand to multiple training sites. Also, the group researched only included resident physicians and would have benefited from the addition of attending physicians. This may have produced contrasting results on those who have already trained in the procedure and possibly performed it on living patients. A survey was conducted using a convenience sample of residents and only included the homemade model. The study could be expanded upon, by comparing the homemade model versus a live tissue model.

## Conclusions

Lateral canthotomy is rarely performed in the emergency department due to its low incidence. Simulation is often needed to train residents in this procedure. Preexisting simulation models such as animals or premade synthetic models have their pitfalls. This "homemade" eye model used in the training of lateral canthotomy proved to be an alternative model for training as our survey validated its effectiveness. Due to its low cost, we will continue to use such a model in our simulation lab for EM residents. It is a good low-cost, low-fidelity alternative to already existing models and encourages other residencies to consider such a model for training.

## References

[1] Kong R, Kaya DP, Cioe-Pena E, Greenstein J (2018). A low fidelity eye model for lateral canthotomy training. Afr J Emerg Med.

[2] Holt GR, Holt JE (1983). Incidence of eye injuries in facial fractures: an analysis of 727 cases. Otolaryngol Head Neck Surg.

[3] Amer E, Abbas AER (2019). Ocular compartment syndrome and lateral canthotomy procedure. J Emerg Med.

[4] (2020). Orbital compartment syndrome. https://eyewiki.org/Orbital_compartment_syndrome.

[5] Voss JO, Hartwig S, Doll C, Hoffmeister B, Raguse JD, Adolphs N (2016). The "tight orbit": incidence and management of the orbital compartment syndrome. J Craniomaxillofac Surg.

[6] Desai NM, Shah SU (2020). Lateral Orbital Canthotomy. https://www.ncbi.nlm.nih.gov/books/NBK557476/.

